# Comparison of chemotherapeutic combination therapy, dosing frequency and dosing sequence for the adjuvant treatment of early-stage breast cancer: a network meta-analysis

**DOI:** 10.1080/07853890.2026.2652096

**Published:** 2026-04-08

**Authors:** Linxiaoxi Ma, Wenjia Zuo, Yizi Zheng, Lichen Tang, Shuling Zhou, Qin Xiao, Lei Fan

**Affiliations:** a Department of Breast Surgery, Fudan University Shanghai Cancer Center, Department of Oncology, Shanghai Medical College, Fudan University, Shanghai, PR China; b Department of Thyroid and Breast surgery, Shenzhen Breast Tumor Research Center for Diagnosis and Treatment, National Standardization Center for Breast Cancer Diagnosis and Treatment, the First Affiliated Hospital of Shenzhen University, Shenzhen Second People’s Hospital, Shenzhen, PR China; c Department of Pathology, Fudan University Shanghai Cancer Center, Shanghai, PR China; d Department of Radiology, Fudan University Shanghai Cancer Center, Shanghai, PR China; e Medical Science Center, Fudan University, Shanghai, PR China

**Keywords:** Adjuvant chemotherapy, breast cancer, cyclophosphamide, network meta-analysis, taxane

## Abstract

**Background:**

Despite the availability of several chemotherapeutic regimens for early-stage breast cancer (BC), the ideal combination and dosing strategy remain to be defined. Thus, we conducted a network meta-analysis (NMA) comparing the current chemotherapeutic regimens used in the treatment of women with early-stage BC.

**Methods:**

We searched public database from inception to May 2021 for Phase II and Phase III trials of adjuvant chemotherapy in patients with early-stage BC following PRISMA guidelines. The primary end-points were event-free survival (EFS) and overall survival (OS) determined by estimates of hazard ratios (HR) and surface under the cumulative ranking curve (SUCRA) values. The safety analysis had only Grade ≥ 3 adverse effects (AEs).

**Results:**

This NMA evaluated a total of 17,187 patients from 36 randomized controlled trials. The follow-up ranged from 22 months to 152 months (median: 12.8 years). The chemotherapeutic regimen AQ (6 T CD + C CD) had the highest SUCRA value (AQ: 0.95). Among the chemotherapeutic regimens, the AF regimen consisting of 4 A CD→T CD showed the highest probability (SUCRA: 0.92) for being the most effective treatment based on the OS. The rank probability assessment revealed that AQD contributed to the lowest incidence of nausea (SUCRA: 0.96), and C (3 CAX CD→TX CD; SUCRA: 0.79) was least likely to induce neutropenia.

**Conclusion:**

This meta-analysis confirmed that concurrent six-cycle treatment with taxane and cyclophosphamide at three-week intervals might be the optimal therapy for treating early-stage BC in the adjuvant setting.

## Introduction

Despite the introduction of targeted therapies for the adjuvant treatment of women with early-stage breast cancer (BC), combination therapy with chemotherapeutic drugs remains the cornerstone of therapeutic management [1]. Unlike targeted therapies, the therapeutic outcome of chemotherapy is not dependent on the expression of hormone receptors or human epidermal growth factor receptor 2 (HER 2) [2]. Hence, cytotoxic chemotherapeutic agents are frequently used to prolong recurrence-free survival in patients with early-stage BC after surgical resection [3]. Among the chemotherapeutic agents, cyclophosphamide, methotrexate, and 5-fluorouracil (CMF)-based regimens were the first to be used for the adjuvant treatment of early-stage BC, followed by the introduction of anthracyclines and taxanes [4].

For early non-metastatic BCs, the primary therapeutic objectives of systemic adjuvant therapy are to prevent local or regional recurrence, to eradicate breast tumors and axillary lymph nodes, to minimize treatment-related adverse effects, and to enhance patients’ quality of life [5]. Based on the expert opinions and several randomized clinical trials (RCTs), the European Society for Medical Oncology (ESMO) and the National Comprehensive Cancer Network (NCCN) clinical practice guidelines recommend a wide array of adjuvant chemotherapy regimens such as the combinations of docetaxel and cyclophosphamide, epirubicin and cyclophosphamide, CMF, and doxorubicin and cyclophosphamide, followed by a taxane and/or capecitabine given either sequentially or concomitantly in dose-dense or conventional manner for the treatment of early-stage BC [6,7].

Taxanes alone or in combination with other chemotherapeutic agents have been used clinically for almost three decades and are considered the most promising first-line treatment option for early-stage BC [8]. Despite the availability of several regimens that provide numerous choices for clinical decision-making, there is a significant lack of direct head-to-head comparisons between these regimens. Moreover, the literature lacks clarity regarding the optimal combination of agents, dosing schedules, and sequencing strategies. This absence of comprehensive comparative data creates challenges for clinicians in making evidence-based decisions, leading to variability in treatment practices. Such gaps underscore the need for a more systematic and integrated analysis to inform clinical guidelines. In this context, a network meta-analysis (NMA) is particularly valuable because it enables indirect comparisons between regimens that have not been compared directly in clinical trials. By synthesizing both direct and indirect evidence, the NMA allows for the ranking of treatment regimens based on effectiveness and safety, providing clinicians with a clearer understanding of the optimal therapeutic strategies for early-stage breast cancer. This analysis aims to fill the critical gaps in the literature by offering a comprehensive assessment of the available chemotherapeutic options, which is currently lacking [9]. Accordingly, to illustrate the ideal combination and dosing frequency of chemotherapeutic agents, we conducted an NMA comparing the contemporary chemotherapeutic regimens used in treating women with early-stage BC.

## Methods

### Data sources and search criteria

A comprehensive systematic literature search was performed in PubMed, Embase/Medline, and Cochrane Library from inception to May 2021 with a predefined search strategy developed using the Population, Intervention, Comparator, and Outcome (PICO) framework. Accordingly, the search strings, which are listed below, included BC and different chemotherapeutic agents with appropriate Boolean operators.

The literature search was implemented with the terms ‘early breast cancer’, ‘cyclophosphamide’, ‘taxanes’, ‘anthracyclines’, ‘docetaxel’, ‘paclitaxel’ ‘methotrexate’, ‘fluorouracil’, ‘doxorubicin’, ‘epirubicin’, ‘dose-dense’, ‘carboplatin’, ‘capecitabine’, ‘gemcitabine’, ‘cisplatin’, ‘carboplatin’, ‘pamidronate disodium’, ‘thiotepa’, ‘ixabepilone’, ‘vinblastine’, ‘vinorelbine’, ‘eribulin mesylate’, and ‘chemotherapy’.

**Figure 1.**
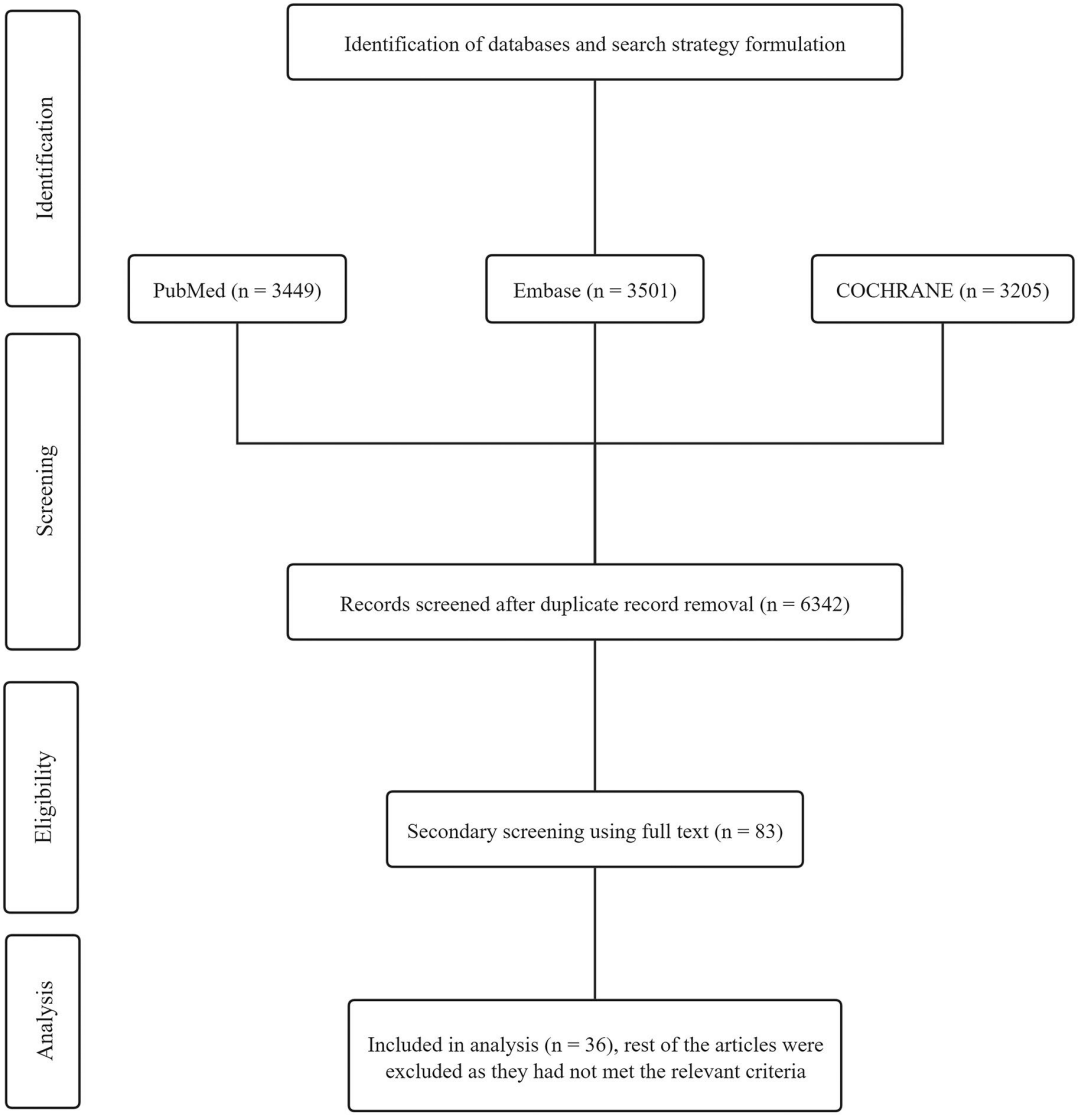
PRISMA flow diagram for inclusion of trials.

The papers included were any randomized controlled trials evaluating the efficacy of chemotherapeutic regimens without targeted or hormonal therapy. Different combinations of chemotherapeutic drugs along with the corresponding dosing frequency (dose-dense vs. conventional dose), dosing schedule (concurrent vs. sequential), and the number of cycles are presented in Figure 1.

The network meta-analysis were performed following Preferred Reporting Items for Systematic Reviews and Meta-Analysis (PRISMA) 2020 framework guidelines.

### Data extraction, selection criteria, and quality assessment

The inclusion and exclusion criteria were prespecified. Eligible trials were any randomized controlled trials evaluating the efficacy of chemotherapeutic regimens without targeted or hormonal therapy. Studies were excluded if they were: (1) non-human studies, letters, reviews, editorial comments or case reports; (2) single-arm or dosage-finding studies; (3) articles without raw data or with a sample size of less than 30; (4) ongoing trials without reported results; (5) All studies included in the analysis were ethically reviewed and approved, and in line with the ethical principles of Declaration of Helsinki on medical research involving human subjects. If several publications from the same trial were identified, only the latest or complete publication was included. Two reviewers independently evaluated the risk of bias for eligible studies using the Cochrane Collaboration risk of bias tool. Any discrepancies were resolved by discussing with all investigators.

One investigator searched, two independent investigator extracted data using a prespecified data-extraction template, which ensured consistency across studies. In the event of discrepancies between the reviewers, a consensus was reached through discussion. If disagreements persisted, a third investigator was consulted to resolve the issue. Two investigator independently evaluated the risk of bias for eligible studies using the Cochrane Collaboration risk of bias too. Articles that met the inclusion criteria were obtained for full-text review and evaluated using a similar process as described above.

The extracted data included information on the number of cycles, dosing schedules, dosing frequency, drug combinations, event-free survival (EFS), overall survival (OS), and adverse events (AEs) of Grade ≥ 3. Cross-checking and quality assurance processes were employed to verify the extracted data before analysis. EFS and OS are the primary outcome measures while safety evaluation by AEs is secondary outcome. Specifically, EFS represents the time to recurrence or any event related to the progression of breast cancer, OS stands for the total length of survival after diagnosis, and AEs mean grade 3 or higher toxicities.

In cases where multiple studies reported the results of the same RCT, only the most recent update with the relevant end-points was included in the final analysis. Studies reporting outcome measures/end-points other than OS, EFS, and AEs, as well as those conducted in neoadjuvant and advanced/metastatic BC settings, were excluded. In addition, non-English articles and studies utilizing targeted therapies were also excluded from this NMA to minimize the skewness of the results. Evaluation of the quality of the included studies used the Cochrane risk-of-bias tool [10,11]. The Cochrane tool has been widely used to assess the risk of bias in randomized controlled trials in systematic evaluations or meta-analyses. It was developed by the Cochrane Collaboration in response to feedback and criticism of the previous Cochrane Risk of Bias tool.The Cochrane tool assesses risk of bias in five areas, including the randomization process, deviations from the intended interventions, missing outcome data, outcome measures, and selection of reported outcomes.

### Network geometry

The evidence gathered for each end-point from the systematic reviews was sorted based on the intervention and comparator regimens. Overall, a total of 25 regimens were present in the final analysis. For brevity and convenience, the regimens were coded alphabetically to simplify the process. The edges signified direct comparisons while the nodes represented different treatment regimens. To arrive at an appropriate network geometry, only the published RCTs were analyzed. For network construction, paclitaxel or docetaxel were considered as taxanes, and epirubicin and doxorubicin as anthracyclines. The extracted treatment regimen information included the number of cycles, dosing schedule, dosing frequency, and drug combinations.

### Statistical analysis

We performed literature deduplication and eligibility assessment of eligibility using Microsoft Excel (Copyright 2020). The statistical software ‘R’ (version: 3.6.1) was used in conjunction with the GeMTC package of RevMan 5.3 to run Bayesian analyses. This package is based on the generalized linear modeling by the Wald test [12]. The inconsistency statistics (*I*2) described the percentage of variation across RCTs [13]. Model fitting for data pooling used convergence between prior and posterior values with deviance information criteria (DIC) [14]. Heterogeneity among the studies was assessed using the *I*2 values and DIC. Effect estimates of hazard ratios (HRs) with 95% confidence intervals (95% CIs) formed the league tables to interpret the results. By plotting rankograms, we calculated the surface under the cumulative ranking curve (SUCRA) values, representing the rank probability of each treatment regimen. SUCRA values are derived from the rank probabilities generated *via* NMA. These probabilities reflect the likelihood of each regimen being ranked at each possible position (first, second, third, etc.). The SUCRA value is then calculated by integrating these probabilities to produce a single measure, which reflects the average ranking of each regimen across all possible ranks. Mathematically, the SUCRA value is the cumulative sum of the rank probabilities up to a given rank, normalized to a scale of 0 to 1, where a SUCRA value of 1 indicates that a regimen consistently ranks as the best across all comparisons. For safety analysis, only AEs of Grade 3 and above (Grade ≥ 3) were analyzed. The severity of AEs was determined by researchers by referring to the General Terminology Standard for Adverse Events (CTCAE) published by the National Cancer Institute (NCI), which classifies AEs into the following five levels: grade 1 (mild), grade 2 (moderate), grade 3 (severe), grade 4 (life-threatening), and grade 5 (AE-associated death).

## Results

### Search results and characteristics of the included studies

The initial electronic search yielded 6,342 consolidated studies from the selected databases, which were further curated to remove duplicates and irrelevant articles during the primary title and abstract screening. A study flow diagram with the results of the search strategy as per the PRISMA guidelines is presented in Figure 1. Following the initial screening, 40 distinct studies were further scrutinized, 36 of which were deemed eligible for our analysis based on the closure of the evidence network. The detailed chemotherapeutic regimens and their letter codes are outlined in Table 1. The baseline demographic profiles of the studies included in the final analysis are presented in Table 2. A total of 17,187 patients were included in the NMA. In brief, five distinct studies had fluorouracil, epirubicin hydrochloride, and cyclophosphamide (FEC) as the comparator regimen [16,17,20,25,32], whereas 20 studies [15,19–21,26,29,33–38,40,41,43–45,47,50] used chemotherapeutic regimens administered as sequential (*→*) treatment in the comparator arm. The follow-up ranged from 22 months to 152 months (median: 12.8 years).

**Table 1.** Chemotherapeutic regimens and the respective letter codes used for generating the network diagram.

Letter coding	Chemotherapy regimen
AA	6 T CD + X
AE	3 FAC CD→Ix CD
AF	4 A CD→T CD
AG	4 A CD→T CD→CMF CD
AJ	4 FAC
AM	6 AC CD + T CD
AO	6 FAC DD
AQ	6 T CD + C CD
AR	8 FAC CD
C	3 CAX CD→TX CD
D	3 FAC CD→T CD
E	4 A CD + T CD
F	4 A CD→CMF CD
H	4 AC CD
J	4 AC CD→T CD
K	4 AC CD→T Gx CD
L	4 AC CD→Ix CD
N	4 AC DD→T DD
P	4 AT CD→X CD
R	4 FAC CD→T CD
S	4 FAC DD→T DD
T	4 T CD→AC CD
V	6 AC CD/CMF CD
X	6 FAC
Y	6 FAC CD
Z	6 T CD + AC CD

*Note*: T, taxanes; A, anthracyclines; FAC fluorouracil-anthracycline- cyclophosphamide; CD, conventional dose; DD, dose-dense; AC, anthracycline-cyclophosphamide; X, capecitabine; CMF, cyclophosphamide-methotrexate-fluorouracil; Gx: gemcitabine; Ix: ixabepilone; →, sequential; +, concurrent.

**Table 2.** Baseline characteristics of the included studies.

SL.No	Author name	Year of publication	Intervention (I) vs Comparator (C)	N (Study)	I (N)	C (N)	Median follow-up	Age			
								Age (median)		Age (Range)	
								I	C	I	C
1	Foukakis et al. [15]	2016	4 EC DD→T DD vs. 3 FEC CD →T CD	2017	1006	1011	5.3 years	51.1	50.3	23.3–69.2	21.4–68.6
2	Blondeaux et al. [16]	2020	6 FEC CD vs. 6 FEC DD	1214	604	610	15.8 years	NA	NA	NA	NA
3	Delaloge et al. [17]	2019	6 T CD + X vs. 6 FEC/FAC	2,832	652	649	5 years	NA	NA	NA	NA
4	Mavroudis et al. [18]	2016	4 FEC DD→T DD vs. 6 T CD + C CD	650	326	324	46 (range, 3.9–76.0) and 47 (range, 3.8–71.0) months	54	59.5	24–75	28–75
5	Lambertini et al. [19]	2019	4 FEC DD→paclitaxel DD vs. 4 FEC CD→P CD	2003	320	132	97 months	52	50	NA	NA
6	Mastro et al. [20]	2015	4 FEC DD→paclitaxel DD vs. 4 FEC CD→P CD	2015	545	500		51	51	43–60	44–59
7	Budd et al. [21]	2014	6 AC DD→P DD vs. 6 AC DD→P DD	2,716	678	697	72 months	50.5	50.7	25–77	23.86
8	Ejlertsen et al. [22]	2017	3 EC CD+T CD vs. 6 C CD+T CD	2,012	1,001	1,011	69 months	NA	NA	NA	NA
9	Minckwitz et al. [23]	2015	6 EC CD/CMF CD vs. 6 nab-P CD + X	400	193	198	22.8 months	NA	NA	NA	NA
10	Perrone et al. [24]	2015	4 CMF CD vs. 4 T CD	299	152	147	70 months	71	71		
11	Polyzos et al. [25]	2010	4 T CD→ EC CD VS. FEC CD	813	378	378	62.5 months	56	57	26–73	28–73
12	Mackey et al. [26]	2012	6 T CD + AC CD vs. 6 FAC CD	1,491	745	746	124 months	49	42–55	49	43–56
13	Dimitrios Mavroudis et al. [27]	2017	4 E CD + T CD vs. 4 E CD→T CD	658	329	329	70.5 months	52	53	28–78	29–76
14	Del Mastro et al. [28]	2015	4 E CD + P CD vs. 6 FEC CD	1,055	535	520	12.8 years	NA	NA	NA	NA
15	V. Moebus et al. [29]	2017	4 EC DD+ P DD vs. 4 EC DD→P DD + X	3,023	1,512	1,511	74 months	50	50	20–71	20–71
16	J.R. Mackey [30]	2016	4 AC CD→ T CD vs. 6 AC CD + T CD	3,298	1,649	1,649	10.5 years	50	50	22–74	24–72
17	Saloustros et al. [31]	2014	4 FEC DD→ P DD vs. 4 FEC DD→ T DD	481	241	240	6 years	55	55	29–75	26–75
18	Kerbrat et al. [32]	2017	6 FEC vs. 4 FEC	1,515	759	756	6.1 years	51	50	27–68	27–66
19	Martin et al. [33]	2015	4 ET CD→ X CD vs. 4 EC CD→ T CD	1,384	715	669	6.6 years	51	51	25–72	27–73
20	Joensuu et al. [34]	2011	3 CEX CD→ TX CD vs. 3 FEC CD→ T CD	1,500	753	747	59 months	52	53	26–65	27–65
21	Coombes et al. [35]	2011	4 E vs. 3 E CD→ T CD	803	397	406	64.7 months	NA	NA	NA	NA
22	Roche et al. [36]	2006	6 FEC CD vs. 3 FEC CD→ T CD	1,999	996	1,003	60 months	50	50	26–67	25–65
23	Ellis et al. [37]	2009	4 FEC CD→T CD vs. 4 E CD→ CMF CD	4,162	2,073	2,089	62	48.9	48.4	SD: 8.6	SD: 8.5
24	Martin et al. [38]	2008	4 AC CD vs. 4 AC CD→P CD	1,246	632	614	66	50	30	24–76	23–76
25	Cameron et al. [39]	2012	4 FEC CD vs. 4 CMF CD	4,391	1,116	1,105	85.6	51.7	51.3	45.8	45.0
26	Earl et al. [40]	2017	4 EC CD→ PGx CD vs. 4 EC CD→ P CD	3,152	1,576	1,576	120	NA	NA	NA	NA
27	Biamco et al. [41]	2006	4 E CD→ CMF CD vs. 4 E CD→T CD →CMF CD	972	486	486	NA	NA	NA	NA	NA
28	Buzdar A et al. [42]	2002	4 FAC CD→P CD vs. 8 FAC CD	524	265	259	60 months	NA	NA	NA	NA
29	Coudert et al. [43]	2012	3 FEC CD→T CD vs. 3 FEC CD→Ix CD	1,999	1,003	996	92.8 months	NA	NA	NA	NA
30	Fountzilas et al. [44]	2005	4 E DD→CMF DD vs. 3 E DD→P DD→CMF DD	600	298	297	62 months	50	50	24–76	22–78
31	Mamounas et al. [45]	2005	4 AC CD vs. 4 AC CD→P CD	3,060	1,531	1,529	64.6 months				
32	Miguel Martin et al. [46]	2013	4 FAC CD→P CD vs. 6 FAC CD	1,925	951	974	5 years	51	50	24–72	24–75
33	Reinisch et al. [47]	2018	3 E DD+P→CMF vs. 4 EC CD→CMF CD	231	113	113	12.3 years	55	55	25–71	32–71
34	Roy C et al. [48]	2012	3 AC CD→P CD vs. 6 AC CD	50	25	25	24 months	46.6	47	18–65	20–66
35	P. Vici et al. [49]	2011	4 EC CD→T CD vs. 4 EC CD	750	374	376	64 months	50	51	43–59	44–60
36	A. Yardley et al. [50]	2017	4 AC CD→Ix CD vs. 4 AC CD→P CD	614	306	308	48 months	53	56	22–80	22–85

Abbreviations: T, taxanes; A, anthracyclines; FAC, fluorouracil-anthracycline-cyclophosphamide; CD, conventional dose; DD, dose-dense; AC, anthracycline- cyclophosphamide; X, capecitabine; CMF, cyclophosphamide-methotrexate-fluorouracil; Gx: gemcitabine; Ix: ixabepilone; →, sequential; +, concurre.

### NMA based on a Bayesian model

#### Efficacy estimates for EFS

**Figure 2.**
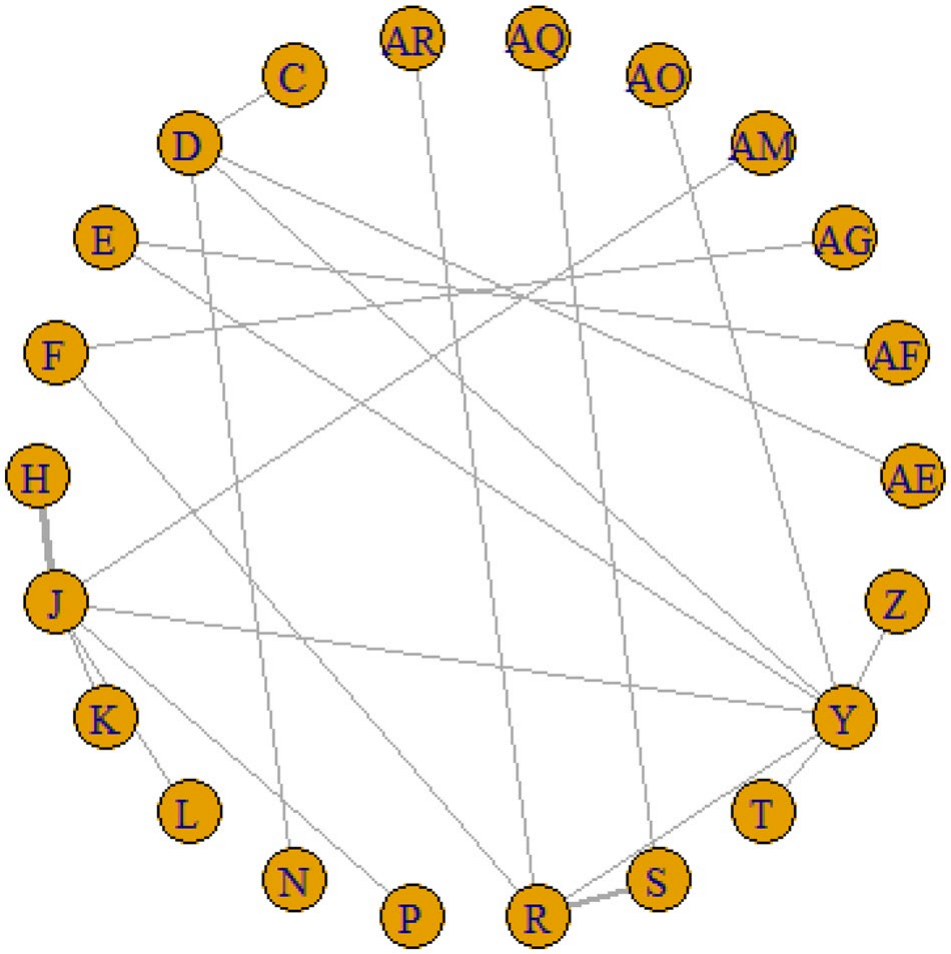
Major network of evidence of the chemotherapeutic regimes for event-free survival.

In terms of EFS, a total of 23 regimens were selected for generating the network of evidence (Figure 2). This network consisted of 23 nodes and 27 edges, of which 7 nodes [(4 AC CD, 4 AC CD→ T CD, 4 AC CD→ TGx CD, 4 AC CD→Ix CD), (4 FAC CD→T CD, 4 FAC DD→T DD), (3 FAC CD →T CD, 3 CAX CD→ TX CD) and (4 T CD→AC CD, 6 FAC CD, Z)] were directly linked, whilst the other 16 nodes were indirectly connected (Figure 2, supplementary Figure 1).

We assessed heterogeneity across studies using the I^2^ statistic, which quantifies the proportion of total variability in the effect estimates attributable to between-study differences. In our analysis, the I^2^ values across the treatment comparisons were generally low (<25%), indicating minimal between-study variability. This suggested that the studies were sufficiently homogenous to justify the use of a fixed-effects model. Additionally, visual inspection of forest plots did not reveal substantial inconsistency between studies, further supporting this decision. Attributing to the DIC and *I*2 values of the fixed- (DIC: 44.81, *I*2: 4%) and random-effects model (DIC: 46.02, *I*2: 4%), data were confirmed to be better suited for the fixed-effects model based on the Bayesian NMA. The rank probability suggested 6 T CD + C CD chemotherapeutic regimen, coded ‘AQ’, as the optimal treatment regimen comparison with the other 23 chemotherapeutic regimens (SUCRA AQ: 0.95), followed by 4 FAC DD→T DD (SUCRA S: 0.94) and 4 A CD→T CD (SUCRA AF: 0.83). The least effective regimen was 4 AT CD→X CD (SUCRA P: 0.02) based on the SUCRA values when analyzed for EFS.

We also calculated HR to compare the treatment effect using HR. In clinical practice, a HR below 1 for a particular chemotherapy regimen suggests that it could be more effective in prolonging recurrence-free survival or reducing mortality compared to the alternatives, making it a more favorable option for patients with specific clinical profiles. Conversely, a HR above 1 may indicate potential limitations or higher risks associated with a regimen, prompting clinicians to weigh these risks against the potential benefits in individual cases. In our analysis, the 6 T CD + C CD (AQ) chemotherapeutic regimen reported a statistically significant HR for EFS as compared to the following list of chemotherapeutic regimens: 3 FAC CD→Ix CD: 0.34 (95% CI: 0.18, 0.64); 4 A CD→T CD→CMF CD: 0.51 (95% CI: 0.29, 0.90); 6 AC CD + T CD: 0.35 (95% CI: 0.18, 0.67); 6 FAC DD: 0.42 (95% CI: 0.2314, 0.78); 8 FAC CD: 0.47 (95% CI: 0.2517, 0.88); 3 CAX CD→TX CD: 0.50 (95% CI: 0.25, 0.99); 3 FAC CD→T CD: 0.39 (95% CI: 0.21, 0.72); 4 A CD + T CD: 0.46 (95% CI: 0.25, 0.86); 4 AC CD: 0.43 (95% CI: 0.22, 0.84); 4 AC CD→T CD: 0.35 (95% CI: 0.18, 0.68); 4 AC CD→T Gx CD: 0.37 (95% CI: 0.19, 0.72); 4 AC CD→Ix CD: 0.38 (95% CI: 0.18, 0.86); 4 AC DD→T DD: 0.50 (95% CI: 0.26, 0.95); 4 AT CD→X CD: 0.27 (0.13, 0.55), and; 6 FAC CD: 0.48 (0.27, 0.86) (Tables 3 and 4). The AQ (6 T CD + C CD) regimen also yielded a better HR compared to all the other treatment regimens, although the values were not statistically significant. Pairwise comparisons are presented in Table 4.

**Table 3.** Rank probability of event-free survival.

Chemotherapeutic regimens	SUCRA rankings
AQ	0.955738
S	0.941881
AF	0.836726
R	0.811571
T	0.795333
F	0.75331
Z	0.742036
N	0.559
C	0.555798
AG	0.533179
Y	0.522762
E	0.456607
AR	0.451738
H	0.41369
AO	0.347536
L	0.302012
D	0.28344
K	0.228536
J	0.182214
AM	0.160571
AE	0.144464
P	0.021857

*Note*: **AE**, 3 FAC CD→Ix CD; **AF**, 4 A CD→T CD; **AG**, 4 A CD→T CD→CMF CD; **AM**, 6 AC CD + T CD; **AO**, 6 FAC DD; **AQ**, 6 T CD + C CD; **AR**, 8 FAC CD; **C**, 3 CAX CD→TX CD; **D**, 3 FAC CD→T CD; **E**, 4 A CD + T CD; **F**, 4 A CD→CMF CD; **H**, 4.

AC CD; **J**, 4 AC CD→T CD; **K**, 4 AC CD→T Gx CD; **L**, 4 AC CD→Ix CD; **N**, 4 AC DD→T DD; **P**, 4 AT CD→X CD; **R**, 4 FAC CD→T CD; **S**, 4 FAC DD→T DD; **T**, 4 T CD→AC CD; **Y**, 6 FAC CD; **Z**, 6 T CD + AC CD.

Abbreviations: T, taxanes; A, anthracyclines; FAC, fluorouracil-anthracycline-cyclophosphamide; CD, conventional dose; DD, dose-dense; AC, anthracycline-cyclophosphamide; X, capecitabine; CMF, cyclophosphamide-methotrexate-fluorouracil; Gx: gemcitabine; Ix: ixabepilone; →, sequential; +, concurrent.

**Table 4.** Hazard ratios and 95% confidence intervals for pairwise comparisons of event-free survival.

AE	0.4679 (0.265, 0.8146)	0.6753 (0.4211, 1.103)	0.9824 (0.673, 1.46)	0.8183 (0.6037, 1.109)	**0.3482 (0.1853, 0.6486**)	0.7381 (0.4233, 1.294)	0.6881 (0.5019, 0.9326)	0.8696 (0.7463, 1.015)	0.7472 (0.5532, 1.017)	0.5437 (0.3618, 0.8175)	0.8022 (0.5496, 1.17)	0.9668 (0.6736, 1.399)	0.9345 (0.6318, 1.381)	0.8908 (0.5075, 1.557)	0.684 (0.5212, 0.9045)	1.252 (0.8043, 1.937)	0.5163 (0.3467, 0.7565)	0.3988 (0.2615, 0.6022)	0.5276(0.36, 0.7637)	0.7141 (0.5618, 0.9061)	0.5688 (0.4316, 0.7574)
2.137 (1.228, 3.773)	AF	1.451 (0.7648, 2.84)	2.105 (1.167, 3.897)	1.743 (1.031, 3.016)	0.7375 (0.346, 1.621)	1.567 (0.7706, 3.243)	1.476 (0.797, 2.68)	1.864 (1.076, 3.218)	1.592 (0.9967, 2.573)	1.162 (0.6394, 2.134)	1.708 (0.952, 3.116)	2.063 (1.158, 3.691)	2.001 (1.111, 3.658)	1.889 (0.913, 3.944)	1.464 (0.8217, 2.621)	2.688 (1.435, 5.004)	1.103 (0.6168, 2.014)	0.8516 (0.4645, 1.601)	1.124 (0.6238, 2.045)	1.516 (0.9145, 2.548)	1.213 (0.7162, 2.068)
1.481 (0.9067, 2.375)	0.6892 (0.3522, 1.308)	AG	1.45 (0.8843, 2.409)	1.203 (0.7714, 1.873)	**0.5124** **(0.294,** **0.9057**)	1.085 (0.6596, 1.81)	1.015 (0.5998, 1.719)	1.285 (0.8123, 2.004)	1.105 (0.6847, 1.72)	0.8004 (0.6227, 1.037)	1.177 (0.7151, 1.946)	1.419 (0.8732, 2.344)	1.381 (0.8302, 2.294)	1.298 (0.6848, 2.511)	1.01 (0.6135, 1.65)	1.844 (1.076, 3.188)	0.7621 (0.5762, 1.011)	0.5865 (0.4266, 0.8123)	0.772 (0.4688, 1.279)	1.052 (0.6967, 1.57)	0.8402 (0.5375, 1.301)
1.018 (0.685, 1.486)	0.475 (0.2566, 0.8567)	0.6896 (0.4152, 1.131)	AM	0.8352 (0.578, 1.184)	**0.3518** **(0.1827,** **0.6759)**	0.7481 (0.415, 1.358)	0.6985 (0.4534, 1.115)	0.8866 (0.6168, 1.27)	0.7605 (0.5303, 1.103)	0.5541 (0.3544, 0.868)	0.8133 (0.6932, 0.958)	0.9802 (0.8699, 1.105)	0.9522 (0.8028, 1.136)	0.9026 (0.5726, 1.41)	0.6953 (0.4588, 1.056)	1.276 (0.982, 1.654)	0.5266 (0.3468, 0.8052)	0.4058 (0.2563, 0.6443)	0.5343 (0.3487, 0.8133)	0.7252 (0.5289, 0.9868)	0.5801 (0.4071, 0.8239)
1.222 (0.9014, 1.656)	0.5738 (0.3316, 0.9702)	0.8314 (0.534, 1.296)	1.197 (0.8449, 1.73)	AO	**0.4242** **(0.2314,** **0.7811)**	0.9022 (0.524, 1.544)	0.8404 (0.5822, 1.231)	1.065 (0.8212, 1.378)	0.9164 (0.6944, 1.195)	0.6638 (0.4581, 0.9761)	0.9769 (0.6892, 1.409)	1.175 (0.8317, 1.658)	1.143 (0.7991, 1.66)	1.078 (0.6261, 1.874)	0.8368 (0.5962, 1.18)	1.535 (1.016, 2.296)	0.6312 (0.4461, 0.9041)	0.4883 (0.3312, 0.7281)	0.6461 (0.4569, 0.9064)	0.8711 (0.7258, 1.043)	0.6984 (0.5507, 0.8835)
2.872 (1.542, 5.397)	1.356 (0.617, 2.89)	1.952 (1.104, 3.401)	2.843 (1.479, 5.472)	2.357 (1.28, 4.322)	AQ	2.11 (1.125, 3.973)	1.988 (1.01, 3.85)	2.501 (1.372, 4.607)	2.163 (1.157, 3.898)	1.569 (0.9414, 2.542)	2.311 (1.18, 4.434)	2.786 (1.461, 5.269)	2.696 (1.388, 5.127)	2.569 (1.152, 5.516)	1.977 (1.044, 3.728)	3.597 (1.808, 7.282)	1.491 (0.9148, 2.407)	1.148 (0.7262, 1.819)	1.522 (0.7981, 2.902)	2.054 (1.159, 3.659)	1.644 (0.9183, 3.012)
1.355 (0.7725, 2.362)	0.6384 (0.3084, 1.298)	0.922 (0.5524, 1.516)	1.337 (0.7365, 2.41)	1.108 (0.6477, 1.908)	**0.4739** **(0.2517,** **0.8886)**	AR	0.9364 (0.5132, 1.699)	1.182 (0.6903, 2.03)	1.024 (0.5817, 1.74)	0.7405 (0.4794, 1.135)	1.092 (0.6057, 1.988)	1.314 (0.7295, 2.35)	1.276 (0.7026, 2.289)	1.211 (0.59, 2.497)	0.933 (0.5225, 1.664)	1.711 (0.9072, 3.168)	0.7042 (0.4633, 1.055)	0.5429 (0.3473, 0.8392)	0.716 (0.3984, 1.302)	0.9674 (0.5811, 1.625)	0.7749 (0.4478, 1.317)
1.453 (1.072, 1.992)	0.6775 (0.3732, 1.255)	0.9857 (0.5818, 1.667)	1.432 (0.8966, 2.206)	1.19 (0.8124, 1.718)	**0.503** **(0.2597,** **0.9901)**	1.068 (0.5884, 1.949)	C	1.262 (0.9609, 1.658)	1.09 (0.7352, 1.579)	0.7912 (0.4989, 1.253)	1.161 (0.7343, 1.773)	1.401 (0.8927, 2.142)	1.361 (0.8654, 2.125)	1.293 (0.6804, 2.337)	0.9937 (0.6933, 1.426)	1.822 (1.075, 2.973)	0.7489 (0.4838, 1.175)	0.5787 (0.3628, 0.9355)	0.7633 (0.4899, 1.19)	1.04 (0.7453, 1.432)	0.828 (0.5728, 1.203)
1.15 (0.9853, 1.34)	0.5363 (0.3107, 0.9292)	0.7779 (0.4989, 1.231)	1.128 (0.7871, 1.621)	0.9386 (0.7259, 1.218)	**0.3998** **(0.2171,** **0.7287)**	0.8458 (0.4925, 1.449)	0.7925 (0.6032, 1.041)	D	0.8626 (0.6564, 1.126)	0.6241 (0.4358, 0.9047)	0.9201 (0.6444, 1.31)	1.108 (0.7948, 1.557)	1.073 (0.7547, 1.551)	1.021 (0.595, 1.768)	0.7886 (0.6281, 0.9934)	1.441 (0.9508, 2.185)	0.5933 (0.4172, 0.847)	0.4582 (0.3089, 0.6819)	0.6059 (0.4255, 0.8501)	0.8203 (0.6843, 0.9827)	0.6541 (0.5177, 0.8345)
1.338 (0.9833, 1.808)	0.628 (0.3887, 1.003)	0.9046 (0.5815, 1.46)	1.315 (0.9065, 1.886)	1.091 (0.8368, 1.44)	**0.4623** **(0.2565,** **0.8643)**	0.9764 (0.5747, 1.719)	0.9174 (0.6335, 1.36)	1.159 (0.8878, 1.524)	E	0.7259 (0.5005, 1.083)	1.071 (0.7366, 1.531)	1.292 (0.9028, 1.808)	1.253 (0.8574, 1.801)	1.189 (0.6904, 2.045)	0.9165 (0.6506, 1.31)	1.672 (1.091, 2.557)	0.6886 (0.4853, 1.013)	0.5321 (0.3599, 0.8065)	0.7043 (0.489, 1.002)	0.9542 (0.7755, 1.172)	0.7622 (0.587, 0.9808)
1.839 (1.223, 2.764)	0.8607 (0.4686, 1.564)	1.249 (0.964, 1.606)	1.805 (1.152, 2.821)	1.506 (1.024, 2.183)	0.6372 (0.3935, 1.062)	1.35 (0.8812, 2.086)	1.264 (0.7982, 2.004)	1.602 (1.105, 2.295)	1.378 (0.9237, 1.998)	F	1.479 (0.9499, 2.286)	1.777 (1.159, 2.724)	1.719 (1.099, 2.701)	1.63 (0.8951, 3.005)	1.252 (0.8272, 1.944)	2.311 (1.433, 3.749)	0.9511 (0.846, 1.07)	0.7346 (0.6017, 0.897)	0.969 (0.6232, 1.512)	1.311 (0.9389, 1.807)	1.047 (0.7169, 1.504)
1.247 (0.8545, 1.82)	0.5854 (0.3209, 1.05)	0.8497 (0.5139, 1.398)	1.23 (1.044, 1.443)	1.024 (0.7096, 1.451)	**0.4327** **(0.2255,** **0.8474)**	0.9159 (0.5031, 1.651)	0.8614 (0.5641, 1.362)	1.087 (0.7631, 1.552)	0.9339 (0.6533, 1.358)	0.6762 (0.4374, 1.053)	H	1.204 (1.088, 1.335)	1.169 (0.9952, 1.375)	1.108 (0.7072, 1.717)	0.8561 (0.5693, 1.308)	1.566 (1.215, 2.024)	0.6443 (0.4235, 0.9912)	0.4987 (0.3128, 0.7935)	0.6558 (0.434, 1.004)	0.8929 (0.656, 1.203)	0.7136 (0.5018, 1.006)
1.034 (0.7145, 1.485)	0.4846 (0.2709, 0.8633)	0.7049 (0.4266, 1.145)	1.02 (0.9048, 1.149)	0.851 (0.6031, 1.202)	**0.3589** **(0.1898,** **0.6846)**	0.7612 (0.4255, 1.371)	0.7136 (0.4668, 1.12)	0.9025 (0.6423, 1.258)	0.774 (0.5532, 1.108)	0.5626 (0.3672, 0.863)	0.8303 (0.7489, 0.9188)	J	0.9712 (0.8584, 1.101)	0.9191 (0.5938, 1.409)	0.7103 (0.4747, 1.062)	1.297 (1.028, 1.642)	0.5358 (0.3561, 0.81)	0.4148 (0.265, 0.6487)	0.5438 (0.3651, 0.8245)	0.7416 (0.5565, 0.9816)	0.5914 (0.4233, 0.8207)
1.07 (0.7242, 1.583)	0.4998 (0.2734, 0.9)	0.7243 (0.436, 1.204)	1.05 (0.8802, 1.246)	0.8746 (0.6024, 1.251)	0.3709 (0.1951, 0.7206)	0.7839 (0.4368, 1.423)	0.7348 (0.4706, 1.155)	0.9318 (0.6448, 1.325)	0.7978 (0.5553, 1.166)	0.5818 (0.3702, 0.9102)	0.8557 (0.7272, 1.005)	1.03 (0.9086, 1.165)	K	0.9463 (0.5976, 1.47)	0.731 (0.4835, 1.124)	1.338 (1.02, 1.732)	0.5525 (0.3602, 0.853)	0.4287 (0.2684, 0.6841)	0.5617 (0.3713, 0.8635)	0.761 (0.5595, 1.045)	0.6082 (0.4275, 0.864)
1.123 (0.6421, 1.971)	0.5294 (0.2536, 1.095)	0.7704 (0.3982, 1.46)	1.108 (0.709, 1.746)	0.928 (0.5335, 1.597)	**0.3893** **(0.1813,** **0.8682)**	0.8256 (0.4004, 1.695)	0.7735 (0.4278, 1.47)	0.9798 (0.5657, 1.681)	0.8411 (0.4889, 1.448)	0.6134 (0.3327, 1.117)	0.9025 (0.5825, 1.414)	1.088 (0.7098, 1.684)	1.057 (0.6804, 1.673)	L	0.7727 (0.4298, 1.388)	1.411 (0.8614, 2.315)	0.5827 (0.3212, 1.06)	0.4493 (0.2411, 0.8429)	0.5967 (0.3306, 1.043)	0.804 (0.4811, 1.332)	0.6446 (0.3739, 1.105)
1.462 (1.106, 1.918)	0.683 (0.3815, 1.217)	0.9905 (0.606, 1.63)	1.438 (0.9469, 2.18)	1.195 (0.8475, 1.677)	**0.5059** **(0.2682,** **0.9583)**	1.072 (0.6011, 1.914)	1.006 (0.7013, 1.442)	1.268 (1.007, 1.592)	1.091 (0.7635, 1.537)	0.7987 (0.5145, 1.209)	1.168 (0.7643, 1.756)	1.408 (0.9416, 2.107)	1.368 (0.8899, 2.068)	1.294 (0.7205, 2.326)	N	1.835 (1.139, 2.916)	0.7556 (0.4985, 1.131)	0.5839 (0.3726, 0.8997)	0.7671 (0.5104, 1.16)	1.041 (0.7775, 1.388)	0.8306 (0.6044, 1.151)
0.7986 (0.5163, 1.243)	0.372 (0.1998, 0.697)	0.5423 (0.3137, 0.9298)	0.7836 (0.6046, 1.018)	0.6516 (0.4354, 0.9845)	**0.278** **(0.1373,** **0.5532)**	0.5844 (0.3157, 1.102)	0.5487 (0.3364, 0.9302)	0.6937 (0.4576, 1.052)	0.5979 (0.3911, 0.9169)	0.4327 (0.2667, 0.6979)	0.6387 (0.4941, 0.8233)	0.771 (0.6092, 0.9732)	0.7473 (0.5774, 0.9803)	0.7089 (0.432, 1.161)	0.5451 (0.3429, 0.878)	P	0.41 (0.2586, 0.6611)	0.3191 (0.1906, 0.5224)	0.4204 (0.2628, 0.6595)	0.5693 (0.3934, 0.8274)	0.4546 (0.3034, 0.6846)
1.937 (1.322, 2.885)	0.9064 (0.4964, 1.621)	1.312 (0.9891, 1.736)	1.899 (1.242, 2.884)	1.584 (1.106, 2.242)	0.6708 (0.4154, 1.093)	1.42 (0.9483, 2.158)	1.335 (0.8512, 2.067)	1.686 (1.181, 2.397)	1.452 (0.9868, 2.061)	1.051 (0.9344, 1.182)	1.552 (1.009, 2.362)	1.866 (1.235, 2.808)	1.81 (1.172, 2.776)	1.716 (0.9438, 3.114)	1.323 (0.8845, 2.006)	2.439 (1.513, 3.866)	R	0.773 (0.6598, 0.9027)	1.017 (0.6708, 1.556)	1.381 (1.014, 1.862)	1.109 (0.7724, 1.533)
2.507 (1.66, 3.824)	1.174 (0.6246, 2.153)	1.705 (1.231, 2.344)	2.464 (1.552, 3.902)	2.048 (1.373, 3.02)	0.8713 (0.5496, 1.377)	1.842 (1.192, 2.879)	1.728 (1.069, 2.756)	2.183 (1.466, 3.237)	1.879 (1.24, 2.779)	1.361 (1.115, 1.662)	2.005 (1.26, 3.197)	2.411 (1.542, 3.774)	2.332 (1.462, 3.726)	2.226 (1.186, 4.148)	1.713 (1.111, 2.684)	3.134 (1.914, 5.247)	1.294 (1.108, 1.516)	S	1.314 (0.8346, 2.089)	1.787 (1.249, 2.514)	1.431 (0.9712, 2.085)
1.895 (1.309, 2.778)	0.89 (0.4891, 1.603)	1.295 (0.7816, 2.133)	1.872 (1.23, 2.868)	1.548 (1.103, 2.189)	0.6569 (0.3445, 1.253)	1.397 (0.7683, 2.51)	1.31 (0.8405, 2.041)	1.65 (1.176, 2.35)	1.42 (0.9983, 2.045)	1.032 (0.6614, 1.605)	1.525 (0.996, 2.304)	1.839 (1.213, 2.739)	1.78 (1.158, 2.694)	1.676 (0.9584, 3.024)	1.304 (0.8622, 1.959)	2.379 (1.516, 3.805)	0.983 (0.6428, 1.491)	0.761 (0.4786, 1.198)	T	1.351 (1.01, 1.819)	1.08 (0.7739, 1.519)
1.4 (1.104, 1.78)	0.6596 (0.3925, 1.094)	0.9508 (0.6371, 1.435)	1.379 (1.013, 1.891)	1.148 (0.9584, 1.378)	**0.4868** **(0.2733,** **0.8631)**	1.034 (0.6156, 1.721)	0.9613 (0.6982, 1.342)	1.219 (1.018, 1.461)	1.048 (0.8529, 1.29)	0.7629 (0.5535, 1.065)	1.12 (0.8313, 1.524)	1.349 (1.019, 1.797)	1.314 (0.9567, 1.787)	1.244 (0.7505, 2.078)	0.9605 (0.7206, 1.286)	1.757 (1.209, 2.542)	0.7239 (0.5372, 0.986)	0.5596 (0.3978, 0.8008)	0.7401 (0.5499, 0.9906)	Y	0.8008 (0.6825, 0.9319)
1.758 (1.32, 2.317)	0.8241 (0.4836, 1.396)	1.19 (0.7689, 1.861)	1.724 (1.214, 2.456)	1.432 (1.132, 1.816)	0.6083 (0.332, 1.089)	1.291 (0.7594, 2.233)	1.208 (0.831, 1.746)	1.529 (1.198, 1.931)	1.312 (1.02, 1.704)	0.955 (0.6648, 1.395)	1.401 (0.9945, 1.993)	1.691 (1.219, 2.362)	1.644 (1.157, 2.339)	1.551 (0.9048, 2.675)	1.204 (0.8691, 1.655)	2.2 (1.461, 3.296)	0.902 (0.6521, 1.295)	0.6987 (0.4796, 1.03)	0.9258 (0.6582, 1.292)	1.249 (1.073, 1.465)	Z

Abbreviations: T, taxanes; A, anthracyclines; FAC, fluorouracil-anthracycline-cyclophosphamide; CD, conventional dose; DD, dose-dense; AC, anthracycline- cyclophosphamide; X, capecitabine; CMF, cyclophosphamide-methotrexate-fluorouracil; Gx: gemcitabine; Ix: ixabepilone; →, sequential; +, concurrent Notes: Values are represented as HR (95% CI).

### Dose-dense vs. conventional dose regimen

Among the 23 regimens included for the analysis of EFS, 3 regimens, namely, 6 FAC DD (AO), 4 AC DD→T DD (N), and 4 FAC DD→T DD (S), administered dose-dense chemotherapeutic drugs. Among them, 4 FAC DD→T DD was the most effective regimen with an HR of 0.773 (95% CI; 0.6598, 0.9027) in comparison to the respective conventional dose regimen (4 FAC CD→T CD). Similarly, 4 AC DD→T DD had a better though not statistically significant HR of 0.7103 (95% CI; 0.4747, 1.062) compared with 4 AC CD→T CD. Whereas the DD regimen without taxanes, 6 FAC DD, was not better than the CD regimen, 6FAC CD (HR:1.148, 95% CI; 0.9584, 1.378) (Table 4).

#### Efficacy estimates for OS

**Figure 3.**
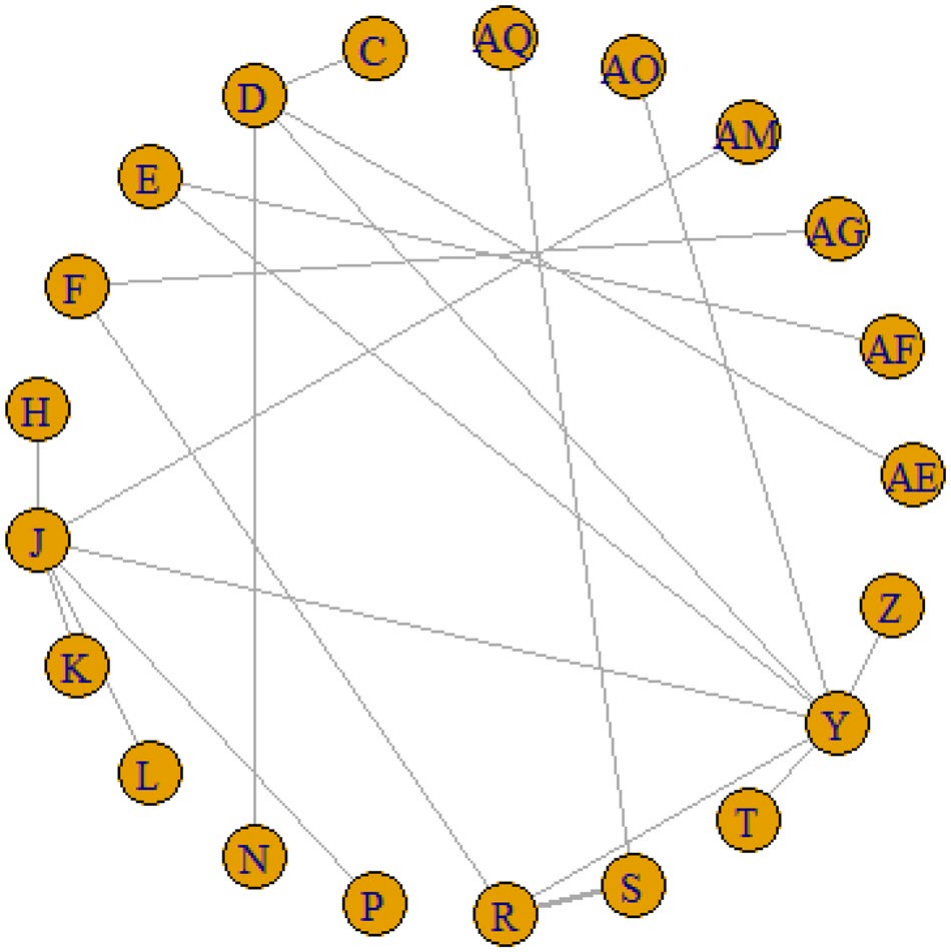
Major network of evidence of the chemotherapeutic regimes for overall survival.

The OS measure of this analysis involved 21 regimens that constituted the network structure shown in Figure 3 and supplementary Figure 2. The *I2* value of 3% was identical in the random-and fixed-effects model, less than 50%, so we performed a Bayesian fixed-effects NMA. The AF regimen consisting of 4 A CD→T CD (Table 5) had the highest probability (SUCRA: 0.92) for being the most effective treatment based on the OS compared to the other regimens, followed by 4 FAC DD→T DD (SUCRA S: 0.91) and 6 T CD + C CD (SUCRA AQ: 0.88).

**Table 5.** Rank probability of overall survival.

Chemotherapeutic regimens	SUCRA rankings
AF	0.919725
S	0.907
AQ	0.883675
Z	0.769875
R	0.675763
F	0.66055
E	0.6449
T	0.54435
H	0.517288
C	0.469925
Y	0.459175
J	0.417275
N	0.413825
K	0.381575
AG	0.375375
AM	0.33665
L	0.331138
AO	0.321225
P	0.276725
D	0.156363
AE	0.037625

*Note*: **AE**, 3 FAC CD→Ix CD; **AF**, 4 A CD→T CD; **AG**, 4 A CD→T CD→CMF CD; **AM**, 6 AC CD + T CD; **AO**, 6 FAC DD; **AQ**, 6 T CD + C CD; **AR**, 8 FAC CD; **C**, 3 CAX CD→TX CD; **D**, 3 FAC CD→T CD; **E**, 4 A CD + T CD; **F**, 4 A CD→CMF CD; **H**, 4 AC CD; **J**, 4 AC CD→T CD; **K**, 4 AC CD→T Gx CD; **L**, 4 AC CD→Ix CD; **N**, 4 AC DD→T DD; **P**, 4 AT CD→X CD; **R**, 4 FAC CD→T CD; **S**, 4 FAC DD→T DD; **T**, 4 T CD→AC CD; **Y**, 6 FAC CD; **Z**, 6 T CD + AC CD.

Abbreviations: T, taxanes; A, anthracyclines; FAC, fluorouracil-anthracycline-cyclophosphamide; CD, conventional dose; DD, dose-dense; AC, anthracycline-cyclophosphamide; X, capecitabine; CMF, cyclophosphamide-methotrexate-fluorouracil; Gx: gemcitabine; Ix: ixabepilone; →, sequential; +, concurrent.

The 4 The A CD→T CD regimen had statistically significant HRs for overall survival compared to 8 FAC CD: 0.25 (95% CI: 0.11, 0.59), 6 FAC DD: 0.39 (0.17, 0.87), 3 FAC CD→T CD: 0.32 (0.14, 0.73), and 6 FAC CD: 0.44 (0.20, 0.97). In addition, the 4 A CD→T CD regimen showed better HRs versus other regimens in the network (Table 6), though not statistically significant. Table 6 shows the pairwise HRs and the 95% CIs of all the chemotherapeutic regimens in the major network for overall survival.

**Table 6.** League table of hazard ratios and 95% confidence intervals of pairwise comparisons of overall survival.

AE	0.2589 (0.1109, 0.5989)	0.6283 (0.3075, 1.24)	0.6567 (0.3423, 1.213)	0.6503 (0.4475, 0.9659)	0.2719 (0.0936, 0.7652)	0.5774 (0.3867, 0.8523)	0.7906 (0.643, 0.9642)	0.4897 (0.3337, 0.7245)	0.4623 (0.2566, 0.8255)	0.5754 (0.2969, 1.113)	0.6205 (0.3283, 1.142)	0.634 (0.3307, 1.197)	0.6771 (0.2989, 1.492)	0.6074 (0.4229, 0.8807)	0.7002 (0.3501, 1.395)	0.4558 (0.2609, 0.8046)	0.3123 (0.1711, 0.5746)	0.5363 (0.3344, 0.8536)	0.5786 (0.4213, 0.8063)	0.428 (0.2917, 0.6236)
3.862 (1.67, 9.017)	AF	2.417 (0.9037, 6.426)	2.509 (0.9532, 6.725)	2.511 (1.139, 5.648)	1.047 (0.2962, 3.661)	2.244 (0.9202, 5.412)	3.05 (1.353, 6.889)	1.896 (0.8999, 3.985)	1.778 (0.716, 4.533)	2.198 (0.8451, 5.973)	2.377 (0.9109, 6.179)	2.423 (0.9411, 6.385)	2.636 (0.9029, 7.562)	2.353 (0.9897, 5.578)	2.72 (0.9856, 7.286)	1.756 (0.7156, 4.533)	1.206 (0.4886, 3.131)	2.062 (0.9052, 4.815)	2.229 (1.03, 4.916)	1.657 (0.7286, 3.652)
1.592 (0.8064, 3.252)	0.4137 (0.1556, 1.107)	AG	1.05 (0.4592, 2.413)	1.037 (0.5456, 1.996)	0.4349 (0.161, 1.122)	0.9265 (0.4336, 1.941)	1.26 (0.6499, 2.458)	0.7791 (0.4077, 1.515)	0.739 (0.5169, 1.052)	0.9204 (0.4002, 2.119)	0.991 (0.4369, 2.226)	1.015 (0.4455, 2.329)	1.09 (0.4129, 2.738)	0.97 (0.4642, 2.053)	1.122 (0.4664, 2.663)	0.7322 (0.4984, 1.069)	0.5034 (0.326, 0.7737)	0.8515 (0.4268, 1.703)	0.9227 (0.4981, 1.707)	0.6813 (0.3583, 1.332)
1.523 (0.8243, 2.922)	0.3986 (0.1487, 1.049)	0.9528 (0.4144, 2.178)	AM	1.002 (0.5599, 1.841)	0.4183 (0.1336, 1.29)	0.8784 (0.4504, 1.784)	1.206 (0.6652, 2.241)	0.7493 (0.4158, 1.389)	0.7119 (0.3437, 1.493)	0.8802 (0.6914, 1.118)	0.946 (0.8133, 1.11)	0.9677 (0.7871, 1.185)	1.04 (0.6042, 1.756)	0.929 (0.4837, 1.863)	1.073 (0.7559, 1.526)	0.703 (0.346, 1.461)	0.4805 (0.228, 1.025)	0.8236 (0.4365, 1.566)	0.8894 (0.5209, 1.548)	0.6561 (0.3691, 1.168)
1.538 (1.035, 2.235)	**0.3982** **(0.1771,** **0.8781)**	0.9639 (0.5009, 1.833)	0.9975 (0.5433, 1.786)	AO	0.4174 (0.1449, 1.191)	0.8816 (0.5509, 1.431)	1.209 (0.8755, 1.688)	0.7496 (0.5538, 1.017)	0.7101 (0.4116, 1.243)	0.8764 (0.4786, 1.616)	0.9484 (0.5297, 1.663)	0.9674 (0.5347, 1.732)	1.04 (0.4737, 2.134)	0.9347 (0.5952, 1.422)	1.071 (0.5539, 2.051)	0.7009 (0.4179, 1.205)	0.4795 (0.2712, 0.8455)	0.8201 (0.5552, 1.205)	0.8837 (0.7306, 1.092)	0.653 (0.5013, 0.866)
3.678 (1.307, 10.68)	0.9553 (0.2731, 3.376)	2.299 (0.8911, 6.21)	2.391 (0.7752, 7.482)	2.396 (0.8395, 6.901)	AQ	2.135 (0.7274, 6.295)	2.912 (1.025, 8.305)	1.805 (0.6385, 5.087)	1.698 (0.7013, 4.183)	2.115 (0.6902, 6.663)	2.274 (0.7414, 7.04)	2.324 (0.7516, 7.271)	2.511 (0.7259, 8.551)	2.242 (0.7743, 6.683)	2.574 (0.8134, 8.097)	1.679 (0.7144, 4.155)	1.152 (0.4999, 2.748)	1.965 (0.6951, 5.714)	2.134 (0.7747, 5.841)	1.562 (0.569, 4.424)
1.732 (1.173, 2.586)	0.4456 (0.1848, 1.087)	1.079 (0.5152, 2.306)	1.138 (0.5604, 2.22)	1.134 (0.6987, 1.815)	0.4684 (0.1589, 1.375)	C	1.371 (0.981, 1.953)	0.8515 (0.5229, 1.392)	0.7975 (0.4201, 1.55)	0.9973 (0.4895, 1.965)	1.075 (0.5362, 2.077)	1.096 (0.5462, 2.147)	1.185 (0.4892, 2.685)	1.055 (0.665, 1.663)	1.217 (0.5787, 2.545)	0.7905 (0.4188, 1.513)	0.5443 (0.2793, 1.075)	0.9252 (0.5446, 1.635)	1.006 (0.6555, 1.555)	0.7401 (0.4718, 1.204)
1.265 (1.037, 1.555)	**0.3279** **(0.1452,** **0.7391)**	0.7936 (0.4069, 1.539)	0.8295 (0.4463, 1.503)	0.8269 (0.5923, 1.142)	0.3433 (0.1204, 0.976)	0.7295 (0.5121, 1.019)	D	0.6217 (0.4419, 0.8682)	0.5863 (0.3343, 1.011)	0.726 (0.3862, 1.358)	0.7808 (0.4258, 1.396)	0.7975 (0.4325, 1.464)	0.8611 (0.392, 1.827)	0.7703 (0.569, 1.041)	0.8852 (0.4465, 1.717)	0.5776 (0.3384, 0.9933)	0.3961 (0.2218, 0.7048)	0.675 (0.4434, 1.031)	0.7319 (0.5699, 0.9479)	0.5419 (0.3936, 0.7457)
2.042 (1.38, 2.997)	0.5275 (0.2509, 1.111)	1.284 (0.6603, 2.453)	1.335 (0.72, 2.405)	1.334 (0.9833, 1.806)	0.5539 (0.1966, 1.566)	1.174 (0.7184, 1.912)	1.608 (1.152, 2.263)	E	0.9417 (0.5535, 1.622)	1.176 (0.6328, 2.128)	1.268 (0.6979, 2.214)	1.293 (0.7083, 2.312)	1.382 (0.6258, 2.919)	1.244 (0.7855, 1.918)	1.433 (0.7332, 2.714)	0.9324 (0.5585, 1.583)	0.641 (0.3676, 1.134)	1.093 (0.7323, 1.631)	1.183 (0.9416, 1.48)	0.8729 (0.6496, 1.177)
2.163 (1.211, 3.897)	0.5623 (0.2206, 1.397)	1.353 (0.9505, 1.935)	1.405 (0.6696, 2.909)	1.408 (0.8042, 2.429)	0.5888 (0.2391, 1.426)	1.254 (0.6454, 2.381)	1.706 (0.9887, 2.991)	1.062 (0.6163, 1.807)	F	1.238 (0.5899, 2.571)	1.333 (0.6406, 2.73)	1.371 (0.6458, 2.818)	1.465 (0.6077, 3.46)	1.318 (0.7071, 2.494)	1.523 (0.6762, 3.351)	0.99 (0.8567, 1.145)	0.6778 (0.5276, 0.8653)	1.159 (0.6331, 2.083)	1.251 (0.7509, 2.045)	0.926 (0.5408, 1.588)
1.738 (0.8981, 3.368)	0.455 (0.1674, 1.183)	1.086 (0.472, 2.499)	1.136 (0.8942, 1.446)	1.141 (0.6187, 2.09)	0.4728 (0.1501, 1.449)	1.003 (0.5088, 2.043)	1.377 (0.7362, 2.59)	0.8505 (0.4699, 1.58)	0.8078 (0.3889, 1.695)	H	1.078 (0.9031, 1.294)	1.104 (0.8804, 1.385)	1.185 (0.6839, 2.033)	1.06 (0.5343, 2.144)	1.221 (0.8519, 1.767)	0.7966 (0.3895, 1.67)	0.5453 (0.2595, 1.16)	0.9442 (0.4849, 1.782)	1.011 (0.5796, 1.776)	0.7457 (0.4092, 1.345)
1.611 (0.8757, 3.046)	0.4208 (0.1618, 1.098)	1.009 (0.4493, 2.289)	1.057 (0.9008, 1.23)	1.054 (0.6012, 1.888)	0.4397 (0.142, 1.349)	0.9299 (0.4816, 1.865)	1.281 (0.7162, 2.348)	0.7887 (0.4518, 1.433)	0.7502 (0.3663, 1.561)	0.9277 (0.773, 1.107)	J	1.021 (0.8924, 1.169)	1.099 (0.6448, 1.814)	0.9782 (0.512, 1.941)	1.135 (0.8289, 1.556)	0.7376 (0.3726, 1.506)	0.5058 (0.244, 1.063)	0.8666 (0.4606, 1.624)	0.937 (0.5544, 1.609)	0.6924 (0.3944, 1.22)
1.577 (0.8355, 3.024)	0.4127 (0.1566, 1.063)	0.9855 (0.4294, 2.245)	1.033 (0.8439, 1.27)	1.034 (0.5773, 1.87)	0.4303 (0.1375, 1.33)	0.912 (0.4658, 1.831)	1.254 (0.6831, 2.312)	0.7734 (0.4325, 1.412)	0.7291 (0.3549, 1.548)	0.9062 (0.7223, 1.136)	0.9791 (0.8557, 1.121)	K	1.079 (0.6208, 1.811)	0.9621 (0.4944, 1.944)	1.112 (0.7892, 1.57)	0.7239 (0.3608, 1.509)	0.4944 (0.2367, 1.047)	0.8485 (0.4461, 1.589)	0.9201 (0.5308, 1.598)	0.6799 (0.3828, 1.219)
1.477 (0.6703, 3.346)	0.3793 (0.1322, 1.108)	0.9175 (0.3652, 2.422)	0.9612 (0.5694, 1.655)	0.9615 (0.4686, 2.111)	0.3982 (0.1169, 1.378)	0.8442 (0.3725, 2.044)	1.161 (0.5472, 2.551)	0.7237 (0.3426, 1.598)	0.6825 (0.289, 1.646)	0.8438 (0.4919, 1.462)	0.9101 (0.5513, 1.551)	0.9264 (0.5521, 1.611)	L	0.8921 (0.3921, 2.071)	1.027 (0.5724, 1.888)	0.672 (0.2901, 1.614)	0.4628 (0.1942, 1.122)	0.7882 (0.3564, 1.778)	0.8512 (0.4189, 1.813)	0.6252 (0.3009, 1.38)
1.646 (1.136, 2.365)	0.425 (0.1793, 1.01)	1.031 (0.4871, 2.154)	1.076 (0.5367, 2.068)	1.07 (0.7034, 1.68)	0.4461 (0.1496, 1.292)	0.9482 (0.6013, 1.504)	1.298 (0.9607, 1.758)	0.8039 (0.5214, 1.273)	0.7585 (0.401, 1.414)	0.9433 (0.4664, 1.872)	1.022 (0.5152, 1.953)	1.039 (0.5143, 2.023)	1.121 (0.4828, 2.55)	N	1.156 (0.5475, 2.395)	0.7482 (0.4004, 1.376)	0.5127 (0.2671, 0.9745)	0.8772 (0.5302, 1.468)	0.9505 (0.6466, 1.406)	0.7052 (0.4624, 1.074)
1.428 (0.717, 2.856)	0.3677 (0.1373, 1.015)	0.8911 (0.3756, 2.144)	0.9319 (0.6554, 1.323)	0.9333 (0.4875, 1.806)	0.3884 (0.1235, 1.229)	0.8214 (0.3929, 1.728)	1.13 (0.5824, 2.239)	0.6976 (0.3684, 1.364)	0.6568 (0.2984, 1.479)	0.819 (0.5659, 1.174)	0.8813 (0.6428, 1.206)	0.8995 (0.6368, 1.267)	0.9735 (0.5297, 1.747)	0.8648 (0.4176, 1.827)	P	0.6494 (0.2989, 1.437)	0.4468 (0.1974, 1.012)	0.7726 (0.3735, 1.523)	0.8276 (0.4444, 1.54)	0.6136 (0.3228, 1.16)
2.194 (1.243, 3.833)	0.5695 (0.2206, 1.398)	1.366 (0.9357, 2.006)	1.423 (0.6844, 2.89)	1.427 (0.83, 2.393)	0.5957 (0.2407, 1.4)	1.265 (0.661, 2.388)	1.731 (1.007, 2.955)	1.073 (0.6316, 1.791)	1.01 (0.8737, 1.167)	1.255 (0.5988, 2.567)	1.356 (0.6641, 2.684)	1.381 (0.6626, 2.771)	1.488 (0.6194, 3.448)	1.336 (0.7265, 2.498)	1.54 (0.696, 3.346)	R	0.6866 (0.5593, 0.843)	1.17 (0.6507, 2.072)	1.265 (0.78, 1.999)	0.9364 (0.5579, 1.555)
3.202 (1.74, 5.844)	0.829 (0.3193, 2.047)	1.986 (1.292, 3.067)	2.081 (0.9752, 4.387)	2.085 (1.183, 3.687)	0.8679 (0.3638, 2)	1.837 (0.9299, 3.58)	2.524 (1.419, 4.509)	1.56 (0.8818, 2.72)	1.475 (1.156, 1.895)	1.834 (0.8622, 3.854)	1.977 (0.9406, 4.098)	2.023 (0.9554, 4.225)	2.161 (0.8911, 5.148)	1.951 (1.026, 3.744)	2.238 (0.9879, 5.066)	1.456 (1.186, 1.788)	S	1.714 (0.9234, 3.104)	1.847 (1.105, 3.102)	1.366 (0.7831, 2.384)
1.865 (1.171, 2.991)	0.485 (0.2077, 1.105)	1.174 (0.5872, 2.343)	1.214 (0.6385, 2.291)	1.219 (0.8298, 1.801)	0.5089 (0.175, 1.439)	1.081 (0.6116, 1.836)	1.482 (0.9696, 2.255)	0.9151 (0.613, 1.366)	0.8629 (0.4801, 1.58)	1.059 (0.5611, 2.062)	1.154 (0.6158, 2.171)	1.179 (0.6292, 2.241)	1.269 (0.5623, 2.806)	1.14 (0.6812, 1.886)	1.294 (0.6564, 2.677)	0.8551 (0.4825, 1.537)	0.5834 (0.3222, 1.083)	T	1.08 (0.7857, 1.5)	0.7989 (0.5475, 1.161)
1.728 (1.24, 2.374)	**0.4486** **(0.2034,** **0.9709)**	1.084 (0.5859, 2.007)	1.124 (0.646, 1.92)	1.132 (0.9154, 1.369)	0.4686 (0.1712, 1.291)	0.9944 (0.643, 1.526)	1.366 (1.055, 1.755)	0.8453 (0.6758, 1.062)	0.7996 (0.4891, 1.332)	0.9891 (0.5631, 1.725)	1.067 (0.6214, 1.804)	1.087 (0.6259, 1.884)	1.175 (0.5516, 2.387)	1.052 (0.711, 1.546)	1.208 (0.6493, 2.25)	0.7902 (0.5002, 1.282)	0.5415 (0.3224, 0.9048)	0.9259 (0.6665, 1.273)	Y	0.7382 (0.6126, 0.9024)
2.336 (1.604, 3.429)	0.6037 (0.2738, 1.372)	1.468 (0.7507, 2.791)	1.524 (0.8561, 2.709)	1.531 (1.155, 1.995)	0.64 (0.226, 1.757)	1.351 (0.8304, 2.12)	1.845 (1.341, 2.541)	1.146 (0.8495, 1.539)	1.08 (0.6297, 1.849)	1.341 (0.7437, 2.444)	1.444 (0.82, 2.535)	1.471 (0.8205, 2.612)	1.599 (0.7247, 3.323)	1.418 (0.9314, 2.163)	1.63 (0.8624, 3.098)	1.068 (0.6429, 1.792)	0.7319 (0.4194, 1.277)	1.252 (0.8614, 1.827)	1.355 (1.108, 1.632)	Z

Abbreviations: T, taxanes; A, anthracyclines; FAC, fluorouracil-anthracycline-cyclophosphamide; CD, conventional dose; DD, dose-dense; AC, anthracycline- cyclophosphamide; X, capecitabine; CMF, cyclophosphamide-methotrexate-fluorouracil; Gx: gemcitabine; Ix: ixabepilone; →, sequential; +, concurrent Notes: Values are represented as HR (95% CI).

#### Safety

**Figure 4.**
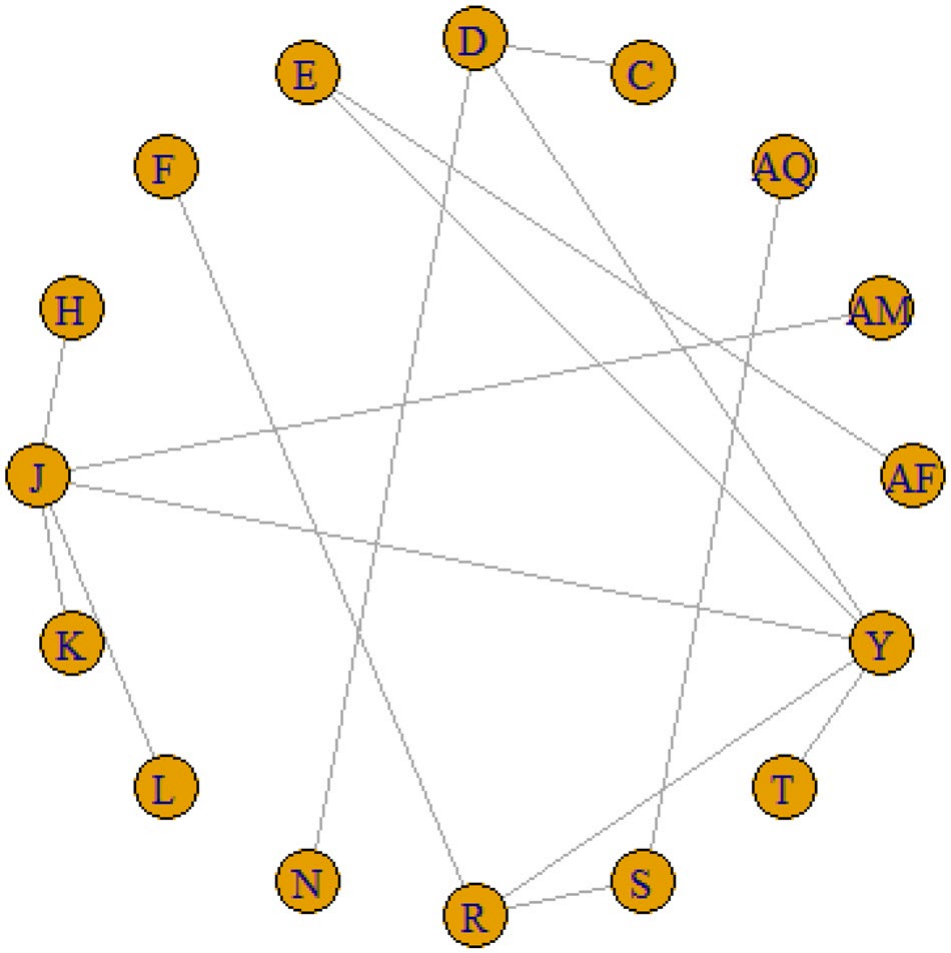
Network for the incidence of Nausea.

The network diagram was completed for only two safety events, namely, nausea and neutropenia, as the network could not be completed for the other Grade ≥ 3 serious AEs. For nausea, the network, comprising 16 nodes and 17 edges, was constructed with 16 chemotherapeutic regimens, of which 9 were directly connected and the rest were indirectly connected (Figure 4, supplementary Figure 3). Since the heterogeneity model based on Bayesian analysis yielded an *I2* value of 10% in both the random- and fixed-effects models, less than 50%, a fixed-effects model was used for analysis.

The rank probability assessed by the SUCRA values showed that 6 T CD + C CD had the lowest incidence of nausea (SUCRA AQ: 0.96), followed by 3 FAC CD →T CD and 4 A CD + T CD with a ranking probability of 0.86. In addition, based on the SUCRA values, the chemotherapeutic regimen with the most frequently occurring Grade ≥ 3 nausea was 4 AC CD→Ix CD (SUCRA L: 0.10), followed by 4 AC CD→T Gx CD (SUCRA K: 0.18). Table 7 lists the rank probabilities of all the 16 chemotherapeutic regimens for the incidence of nausea.

**Table 7.** Rank probability for the incidence of nausea.

Chemotherapeutic regimens	SUCRA rankings
AQ	0.964367
D	0.864817
E	0.863367
AF	0.731867
S	0.648467
Y	0.584417
R	0.4969
C	0.4407
N	0.397867
J	0.39585
F	0.386717
T	0.35495
H	0.306483
AM	0.2703
K	0.18685
L	0.106083

*Note*: **AF**, 4 A CD→T CD; **AM**, 6 AC CD + T CD; **AQ**, 6 T CD + C CD; **C**, 3 CAX CD→TX CD; **D**, 3 FAC CD→T CD; **E**, 4 A CD + T CD; **F**, 4 A CD→CMF CD; **H**, 4 AC CD; **J**, 4 AC CD→T CD; **K**, 4 AC CD→T Gx CD; **L**, 4 AC CD→Ix CD; **N**, 4 AC DD→T DD; **R**, 4 FAC CD→T CD; **S**, 4 FAC DD→T DD; **T**, 4 T CD→AC CD; **Y**, 6 FAC CD.

Abbreviations: T, taxanes; A, anthracyclines; FAC, fluorouracil-anthracycline- cyclophosphamide; CD, conventional dose; DD, dose-dense; AC, anthracycline-cyclophosphamide; X, capecitabine; CMF, cyclophosphamide-methotrexate-fluorouracil; Gx: gemcitabine; Ix: ixabepilone; →, sequential; +, concurrent.

**Figure 5.**
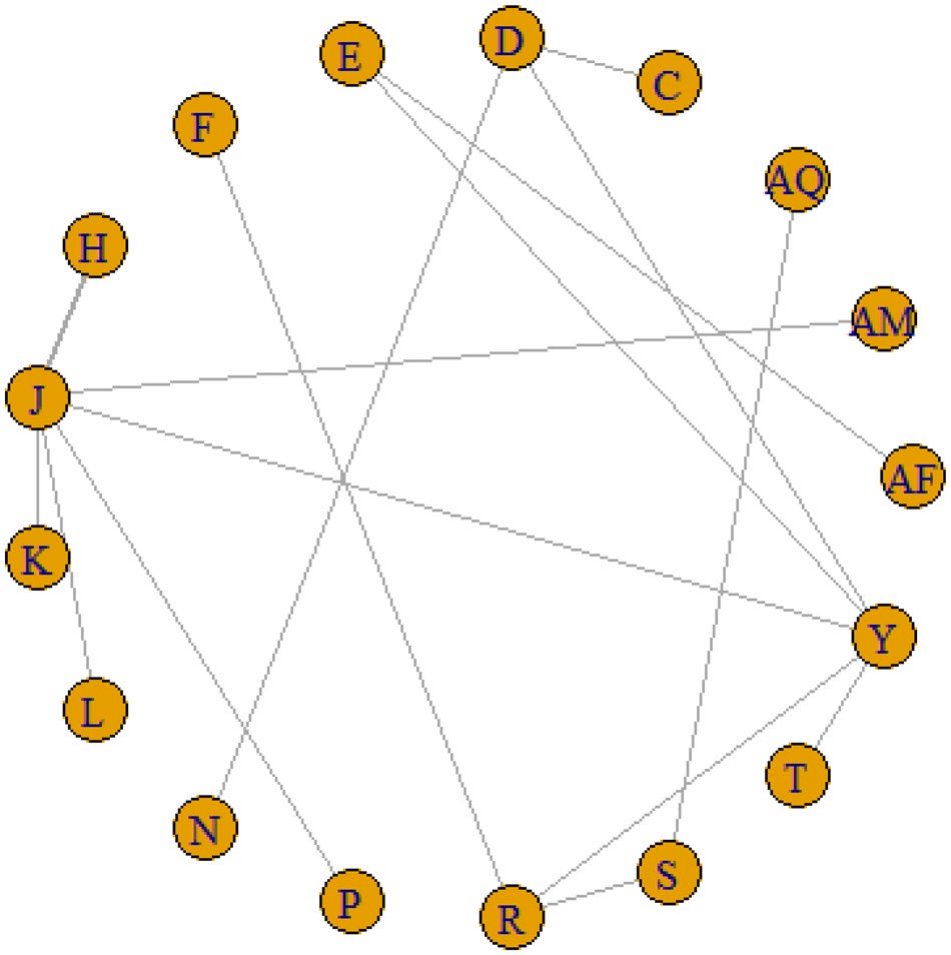
Network for the incidence of neutropenia.

For neutropenia, the *I^2^* value in the random-effects model (3%) was less than the value obtained in the fixed-effects model (34%), so a Bayesian random-effects NMA was performed for this AE. A total of 17 chemotherapeutic regimens were included for synthesizing the evidence network (17 nodes and 22 edges), 12 of which were directly connected and the rest were indirectly connected (Figure 5, supplementary Figure 4).

We found that the regimen 3 CAX CD→TX CD (SUCRA C: 0.79) was least likely to induce neutropenia, followed by 3 FAC CD→T CD (SUCRA D: 0.78) and 4 AC DD→T DD (SUCRA N: 0.76) (Table 8). The treatment regimens that had the highest probability of causing Grade ≥ 3 neutropenia was 6 T CD + C CD (SUCRA AQ: 0.09), followed by 4 AC CD→Ix CD (SUCRA L: 0.10).

**Table 8.** Rank probability for the incidence of neutropenia.

Chemotherapeutic regimens	SUCRA rankings
F	0.654125
C	0.797453
D	0.783984
N	0.765531
E	0.665016
P	0.63125
R	0.631016
AF	0.625313
Y	0.576641
J	0.371031
T	0.36925
H	0.362031
AM	0.361906
S	0.312641
K	0.290578
L	0.212453
AQ	0.089781

*Note*: **AF**, 4 A CD→T CD; **AM**, 6 AC CD + T CD; **AQ**, 6 T CD + C CD; **C**, 3 CAX CD→TX CD; **D**, 3 FAC CD→T CD; **E**, 4 A CD + T CD; **F**, 4 A CD→CMF CD; **H**, 4 AC CD; **J**, 4 AC CD→T CD; **K**, 4 AC CD→T Gx CD; **L**, 4 AC CD→Ix CD; **N**, 4 AC DD→T DD; **R**, 4 FAC CD→T CD; **S**, 4 FAC DD→T DD; **T**, 4 T CD→AC CD; **Y**, 6 FAC CD.

Abbreviations: T, taxanes; A, anthracyclines; FAC, fluorouracil-anthracycline-cyclophosphamide; CD, conventional dose; D, dose-dense; AC, anthracycline-cyclophosphamide; X, capecitabine; CMF, cyclophosphamide-methotrexate-fluorouracil; Gx: gemcitabine; Ix: ixabepilone; →, sequential; +, concurrent.

## Discussion

Given the availability of several chemotherapeutic regimens for the treatment of BC at different doses and at varying frequencies, it is difficult to determine the optimum adjuvant chemotherapy regimen. Conventionally, dose-dense regimens were considered to be advantageous over conventional dose regimens. The results of this NMA revealed that the DD regimen containing taxanes was more effective than the one without taxanes. A previous patient-level NMA also revealed similar results [2].

The results of this NMA provide many noteworthy conclusions. The regimens AQ (6 cycles of taxanes CD plus cyclophosphamide CD), S (4 cycles of fluorouracil-anthracycline- cyclophosphamide DD sequential Taxane DD), and AF (4 cycles of anthracycline CD with sequential cyclophosphamide-methotrexate-fluorouracil CD) may be prioritized for the treatment of early-stage BC in the general population based on extended EFS and OS and a lower risk of AEs.

In 2015, Fujii et al. conducted an NMA of adjuvant BC therapies that compared the efficacy of sequential anthracycline-cyclophosphamide regimens followed by a taxane to TC (docetaxel and cyclophosphamide) for four cycles, TAC (concurrent docetaxel, doxorubicin, and cyclophosphamide), AC (four cycles doxorubicin and cyclophosphamide every three weeks), CMF, platinum agents, and no chemotherapy [51]. This study could not report a statistically significant difference in disease-free survival (DFS) and OS when comparing the CA-T group to TAC, platinum-containing regimens, and four cycles of TC [51]. Another NMA also demonstrated similar efficacy across DACT (dose-dense doxorubicin and cyclophosphamide every two weeks for four cycles), ACWKT (doxorubicin plus cyclophosphamide every three weeks for four cycles followed by weekly paclitaxel), TAC, and four cycles of TC [52]. Although these studies produced valuable findings, they only analyzed TC for four cycles versus TC for six cycles as studied in the ABC trial and the WSG PlanB trial [53,54]. They assumed similar efficacy between the four- and six-cycle TC regimens, based on a 2012 study by the Cancer and Leukemia Group, reporting no differences in DFS and OS between four-cycle AC versus six-cycle AC for early-stage BC [55].

Moreover, the HRs of EFS and OS in the AQ (6 T CD + C CD) and S (4 FAC DD→T DD) regimens derived from this NMA are consistent with an earlier multicenter RCT conducted by the Hellenic Oncology Research Group (HORG) using analogous regimens in women with HER2-negative, axillary lymph node-positive early BC. This study elucidated the non-inferiority of the six-cycle docetaxel and cyclophosphamide regimen conducted every 21 days to the four-cycle dose-dense 5-fluorouracil-epirubicin-cyclophosphamide performed every two weeks followed by four cycles of docetaxel every two weeks, demonstrating similar 3-year DFS rates in both treatment arms [18]. However, a pooled analysis of invasive BC by the same HORG failed to establish the non-inferiority of the aforementioned taxane-cyclophosphamide regimen (DFS: 89.04%; 95% CI: 88%, 90%) and taxane-anthracycline-cyclophosphamide regimen (DFS: 90.32%; 95% CI: 89%, 91%). Though the absolute difference in the DFS was 1.28%, the overall toxicity profile of the anthracycline-free (taxane-cyclophosphamide) regimen was comparatively better [56].

Despite the availability of previous NMAs, this NMA is, to the best of our knowledge, the first to compare the dosing schedules, dosing frequencies, and dosing combinations of chemotherapeutic agents used for the adjuvant treatment of early-stage BC. Still, this study shows some limitations. First, the studies included in this NMA, spanning almost two decades (2002–2020), were conducted at different sites in different demographics, making it impossible to quantify the variations in the efficacy of radiotherapy and surgical interventions. However, it is also a global perspective, providing a systematic assessment on a global scale, covering different geographic regions and populations. Second, clinical heterogeneity in patient characteristics associated with the risk of recurrence could be an important confounding factor. Moreover, some other factors should be taken into consideration when interpreting the results of this study, such as the classification of paclitaxel and docetaxel as taxanes and epirubicin and doxorubicin as anthracyclines when synthesizing the network evidence. Third, the limited number of trials for some drug combinations weakened the reliability of the comparisons. Besides, our study lacked access to individual patient data, hindering our ability to identify patients who could benefit from less intensive treatment. Fourth, the inconsistent availability of time-to-event data for neoadjuvant therapies restricts our ability to link treatment plans with survival advantages. Lastly, slight variations in how different trials defined DFS and EFS and our combined analysis of these outcomes might have introduced additional heterogeneity and potential bias.

## Conclusion

Based on the available evidence, six cycles of taxanes every three weeks combined with cyclophosphamide provide a better EFS and the lowest incidence of nausea compared to the other contemporary adjuvant chemotherapies for patients with early-stage BC.

## Supplementary Material

Supplementary Figure 2.tif

PRISMA_2020_checklist.docx

Supplementary Figure 1-300dpi.tif

Supplementary Figure 2-300dpi.tif

Supplementary Figure 1.tif

Supplementary Figure 3-300dpi.tif

Supplementary Figure 3.tif

Supplementary Figure 4-300dpi.tif

Supplementary Figure 4.tif

## Data Availability

The authors confirm that data supporting the findings of this study are available within the article.
